# *α*-Lipoic Acid Increases Collagen Synthesis and Deposition in Nondiabetic and Diabetic Rat Kidneys

**DOI:** 10.1155/2021/6669352

**Published:** 2021-03-11

**Authors:** Nevena Grdović, Jovana Rajić, Jelena Arambašić Jovanović, Svetlana Dinić, Anja Tolić, Miloš Đorđević, Marija Đorđević, Svetlana Trifunović, Melita Vidaković, Aleksandra Uskoković, Mirjana Mihailović

**Affiliations:** ^1^Department of Molecular Biology, Institute for Biological Research “Siniša Stanković, ” National Institute of Republic of Serbia, University of Belgrade, Bulevar Despota Stefana 142, 11060 Belgrade, Serbia; ^2^Department of Cytology, Institute for Biological Research “Siniša Stanković, ” National Institute of Republic of Serbia, University of Belgrade, Bulevar Despota Stefana 142, 11060 Belgrade, Serbia

## Abstract

*α*-Lipoic acid (ALA) is widely used as a nutritional supplement and therapeutic agent in diabetes management. Well-established antioxidant and hypoglycemic effects of ALA were considered to be particularly important in combating diabetic complications including renal injury. The present study evaluated the potential of ALA to affect profibrotic events in kidney that could alter its structure and functioning. ALA was administered intraperitoneally (10 mg/kg) to nondiabetic and streptozotocin-induced diabetic male Wistar rats for 4 and 8 weeks. The effects of ALA were assessed starting from structural/morphological alterations through changes that characterize profibrotic processes, to regulation of collagen gene expression in kidney. Here, we demonstrated that ALA improved systemic glucose and urea level, reduced formation of renal advanced glycation end products (AGEs), and maintained renal structural integrity in diabetic rats. However, profibrotic events provoked in diabetes were not alleviated by ALA since collagen synthesis/deposition and expression of transforming growth factor-*β*1 (TGF-*β*1) and *α*-smooth muscle actin (*α*-SMA) remained elevated in ALA-treated diabetic rats, especially after 8 weeks of diabetes onset. Moreover, 8 weeks treatment of nondiabetic rats with ALA led to the development of profibrotic features reflected in increased collagen synthesis/deposition. Besides the TGF-*β*1 downstream signaling, the additional mechanism underlying the upregulation of collagen IV in nondiabetic rats treated with ALA involves decreased DNA methylation of its promoter that could arise from increased Tet1 expression. These findings emphasize the therapeutic caution in the use of ALA, especially in patients with renal diabetic complication.

## 1. Introduction

Diabetic kidney disease (DKD) or diabetic nephropathy is one of the microvascular complications with incidence of 20-40% among diabetic patients which is accompanied with progressive structural alterations and loss of kidney functions [1]. Pathophysiological mechanism that underlies DKD is complex and starts with chronic hyperglycemia that in turn triggers oxidative stress, formation of advanced glycation end products (AGEs), activation of MAPK/PKC signaling pathways, and subsequent expression of several proinflammatory and profibrotic mediators [2, 3]. Crosstalk between these events results in the accumulation of extracellular matrix (ECM) proteins in the mesangium and tubulointerstitium with classical histopathological pattern of renal fibrosis. It starts with the structural changes such as thickening of basement membrane and mesangial expansion, gradually progressing into glomerulosclerosis and interstitial fibrosis eventually culminating towards renal failure [4, 5].

Transforming growth factor-*β*1 (TGF-*β*1) downstream signaling is a part of wound healing process that, upon sustained inflammation, hyperglycemia, or oxidative stress that characterize diabetes, converts into pathophysiological fibrosis [6]. TGF-*β*1 is a central mediator of DKD pathogenesis being a main regulator of ECM protein expression in patients with diabetes, probably as a result of hyperglycemic and disturbed oxidative environment [7–10]. TGF-*β*1 induces ECM accumulation by stimulation of type I and IV collagen, *α*-smooth-muscle actin (*α*-SMA), laminin, and fibronectin synthesis, as well as by inhibition of ECM-degrading enzymes [11].

Collagen type IV is the most abundant constituent of the basement membrane [12], expressed in glomerular and tubular basement membranes under physiological conditions [13]. However, diabetic nephropathy is accompanied with an aberrant expression of collagen type IV which includes excessive production and irregular distribution through interstitium and mesangium [14–16]. Among six isoforms of type IV collagen (*α*1-*α*6), *α*1(IV) and *α*2(IV) are universally present in all basement membranes. Collagen *α*1(IV) and *α*2(IV) chains are encoded by *Col4a1* and *Col4a2* genes whose transcriptional regulation relays on well-described *cis-trans* interactions [17], but recent findings highlight the important role of epigenetic mechanisms, including DNA methylation, in the regulation of their expression [18–20].

In view of pathophysiological mechanisms that lie behind DKD, treatment strategies are straightforward and targets hyperglycemia and oxidative stress. The hypoglycemic and antioxidant potential of ALA, which is one of the widely used antioxidants, is found to ameliorate renal pathological changes associated with diabetic nephropathy [21–23]. However, related to skin aging, the beneficial effects of ALA through increased collagen type I synthesis in human dermal fibroblasts were reported [24]. Considering pathophysiology of DKD, similar effect of ALA could be detrimental for diabetic patients with renal complications. With this background, the present study was focused to evaluate the effects of ALA on renal structural/morphological features and profibrotic processes with emphasis to collagen type IV gene expression in the diabetic and nondiabetic rats.

## 2. Material and Methods

### 2.1. Animals, Treatments, and Experimental Setup

All experiments were performed on 2.5-month old male albino Wistar rats weighing between 220 and 250 g. All animal procedures approved by the Ethical Committee for the Use of Laboratory Animals of the Institute for Biological Research “Siniša Stanković,” University of Belgrade were in compliance with the Directive 2010/63/EU on the protection of animals used for experimental and other scientific purposes. The animals in this study were sacrificed by a physical method of euthanasia using a guillotine, according to all recommendations of the guidelines for euthanasia of animals. Death ensues instantly and animals did not suffer. Euthanasia was performed in the operation room, separated from the housing room, one animal by one (the rest of the animals in the experiment were held in experimental room which is separated from the operation room where euthanasia was performed to avoid stress for the other animals). Euthanasia was performed by well-trained and experienced stuff. The equipment used for decapitation was in good working order (services are on a regular basis to provide sharpness of the blades).

Diabetes was induced by multiple low-dose (40 mg/kg) injections of streptozoticin (STZ) (MP Biomedicals) for five consecutive days; control rats were injected with the vehicle alone (0.1 M sodium citrate buffer, pH 4.5). Blood glucose level was measured 24 h after the last STZ injection with a blood Accu-Chek Active glucometer, and the animals were considered as diabetic when the fasting blood glucose level exceeded 20 mmol/l. The rats were randomly divided into four experimental groups: control (control, *n* = 8), control treated with ALA (ALA, *n* = 8), diabetic (STZ, *n* = 10), and diabetic group treated with ALA (STZ/ALA, *n* = 10). ALA (Ivančić i sinovi, Belgrade, Serbia) was injected intraperitoneally (10 mg/kg) every day for 4 and 8 weeks, starting from the last STZ injection. After sacrifice and blood collection, an abdominal incision was made and both kidneys were removed. One kidney was frozen in liquid nitrogen, kept at -80°C, and used for protein, DNA, and RNA isolation, while the other was fixed in 10% buffered formalin for histological examinations.

### 2.2. Biochemical Analysis

Rats were fasted overnight before sacrifice. Blood serum was collected after blood clotting and centrifugation at 2,000 g for 10 min. The serum was used for determination of glucose concentrations with a commercial kit (Gluco-quant Glucose/HK; Boehringer), serum triglycerides level by the GPO-PAP method using an enzymatic kit (Randox Laboratories, UK), urea level by Urea Assay Kit (Abcam, USA), and creatinine concentrations using Cayman's Creatinine Assay according to the manufacturer's instruction.

### 2.3. Histological Analysis and Immunostaining

Formalin-fixed kidney tissues were dehydrated in a decreasing series of xylol and alcohols for posterior inclusion in paraffin blocks. Tissue blocks were sectioned at 5 *μ*m on a rotary microtome (RM 2125RT; Leica Microsystem). In order to observe changes in renal morphology, tissue sections were stained using the Periodic Acid–Schiff (PAS) method. Further, stained sections were analyzed using Olimpus microscope (BX-51) equipped with a microcator, motorised stage, and camera, controlled by the newCAST stereological software package Visiopharm Integrator System. The tubular lumen areas were obtained at objective magnification 40x and expressed as percentage (%) per examined area. Mesangial matrix fraction was evaluated as the area of positive PAS staining, expressed as % per total glomerular area (objective magnification 100x).

For collagen fiber detection, tissue sections were stained with Masson trichrome stain. After staining, sections were observed under a light microscope (DM RB Photomicroscope Leica Microsystems GmbH, Wetzlar, Germany, equipped with Leica DFC 320 CCD camera; objective magnification 40x). Image analysis was done by ImageJ software (version 1.52p, National Institutes of Health). Images were converted to RGB stacks and manual thresholding was applied to evaluate proportion of collagen area per tissue area.

Immunohistochemical analysis was performed as described in [25] using polyclonal antibody raised against N(𝜀)-(carboxymethyl)lysine (CML) (dilution 1 : 50) (Santa Cruz Biotechnology, CA, USA) and secondary antibody horseradish peroxidase (1 : 100) (Santa Cruz Biotechnology). Tissue sections were stained with 3,3′-diaminobenzidine (DAB), counterstained with haematoxylin, and mounted and observed under a light microscope (Leica DM RB Photomicroscope; objective magnification 40x). Image analysis was done by ImageJ software (version 1.52p, National Institutes of Health). Images were processed by color deconvolution with H DAB vector option. CML signaling was calculated as mean grey intensity of DAB staining normalized to the number of nuclei (obtained using Analyze Particles option).

### 2.4. Western Blot Analysis

25 *μ*g of kidney homogenates were separated by SDS–PAGE, transferred onto polyvinylidene difluoride membranes, and probed with polyclonal antibody against GAPDH (FL-335) and monoclonal antibodies against rat TGF-*β*1 (3C11) and *α*SMA (CGA7) all from Santa Cruz Biotechnology and all diluted in 1 : 1,000 ratios. Detection was performed by an enhanced chemiluminescence detection system (Santa Cruz Biotechnology). Quantification of the immunoreactive bands was performed using TotalLab (Phoretix) electrophoresis software (version 1.10) and normalized to the GAPDH used as a loading control.

### 2.5. Real-Time Quantitative PCR (RT-qPCR)

Total RNA was isolated from kidneys using the RNeasy Mini Kit (Qiagen). For cDNA synthesis, 1 *μ*g of the total RNA was treated with DNase I and reverse-transcribed with the RevertAid First-Strand cDNA Synthesis Kit (Fermentas, Burlington, Canada) using mix of random hexamer and oligo (dT) primers. Levels of mRNA were quantified using 2x Maxima SYBR Green/ROX qPCR Master Mix (Fermentas) on a QuantStudio 3 Real-Time PCR system (Applied Biosystems, Carlsbad, CA, USA). Primer-BLAST (https://www.ncbi.nlm.nih.gov/tools/primer-blast/) was used to design the primers (listed in Supplementary table S1) for assessment of *Col4a1*, *Dnmt1*, *Dnmt3a*, *Dnmt3b*, and *Tet1* gene expressions. The thermal cycles consisted of initial denaturation at 95°C/10 min and 40 cycles of two-step PCR at 95°C/15 s and 60°C/60 s. The mRNA expression levels of the genes of interest were normalized to the mRNA expression level of the *Actb* as a reference gene by the 2^-dCT^ method.

### 2.6. Genomic DNA Isolation and Bisulfite Conversion of DNA

Genomic DNA was isolated using Bio-On-Magnetic-Beads (BOMB) approach [26], according to protocol for DNA extraction from mammalian tissue (available online at https://bomb.bio/protocols/). Genomic DNA was subjected to BOMB bisulfite conversion protocol (available online at https://bomb.bio/protocols/) [26].

### 2.7. Measurement of Global Levels of DNA Methylation

Global DNA methylation levels were measured using the 5-mC DNA ELISA kit (Zymo Research, California, USA) according to the manufacturer's instructions. Purified genomic DNA (100 ng) isolated from kidneys from all experimental groups was used, and each sample was assayed in duplicate. Statistical significance was estimated using one-way ANOVA with blocking, treating each ELISA plate as a block.

### 2.8. Methylation-Specific PCR (MSP)

Primer pairs for MSP analysis, designed on promoter region of *Col4a1* encompassing transcription start site (TSS), were designed using MethPrimer (http://www.urogene.org/cgi-bin/methprimer/methprimer.cgi) (Supplemetary table S2). Positions of primers (relative to TSS for *Col4a1*, marked as +1) used for MSP analysis are as follows: M fw (-35, -14), M rev (+44, +64), U fw (-35, -14), and U rev (+45, +69). As each primer covers 2 CpGs, 4 CpGs in total were tested for methylation status per primer pair. The reaction mixture for MSP contained Maxima SYBR Green/ROX qPCRqRT-PCR Master Mix (Fermentas), 20 ng of bisulfite converted DNA, and 0.5 *μ*M of each primer specific for methylated (M) and unmethylated (U) DNA at a final volume of 10 *μ*L. MSP was conducted on the QuantStudio 3 Real-Time PCR system (Applied Biosystems). PCR cycling conditions were as follows: initial denaturation step at 95°C/10 min and followed by 40 cycles of denaturation at 95°C/15 s, and annealing and elongation at 58°C/60 s. The relative level of methylated DNA was expressed using the methylation index defined as: 2 ^(demethylated cycle Ct) − (methylated cycle Ct)^ [27].

### 2.9. Statistical Analysis

The data were expressed as the mean ± SD (standard deviation) for all parameters studied. Student's *t*-test was used to compare means of normally distributed variables between two groups. Statistical differences between groups were analyzed using one-way analysis of variance (ANOVA), followed by Tukey multiple comparison test using GraphPad Prism Software, ver. 5.00 (GraphPad Software, San Diego, CA, USA).

## 3. Results

### 3.1. Effect of ALA on Biochemical Indices in Diabetic Rats

**Figure 1:**
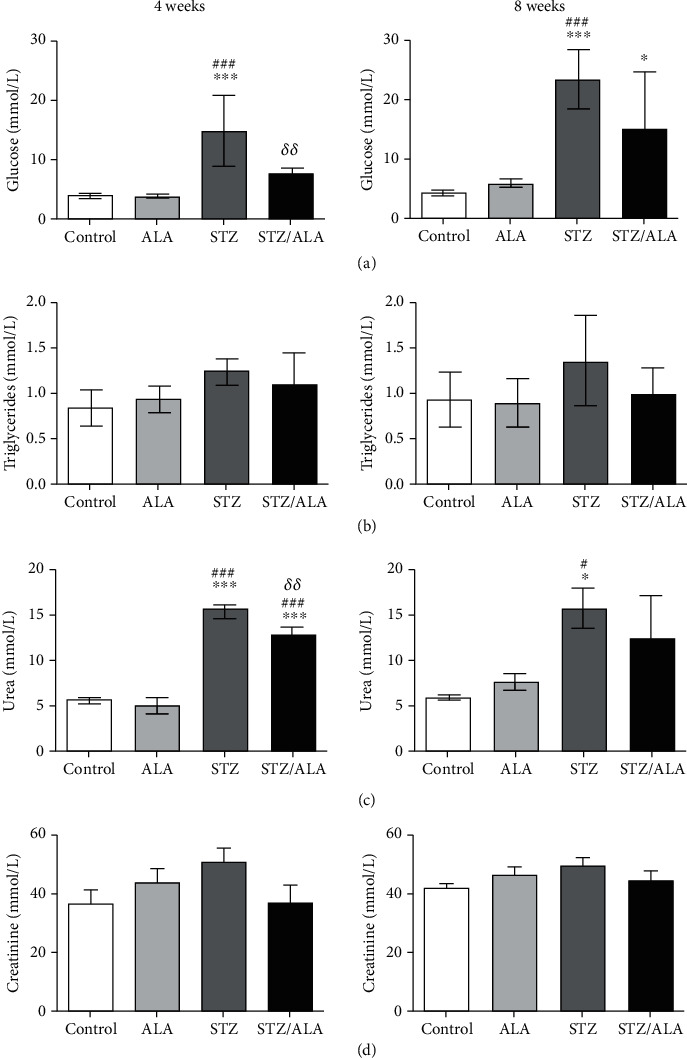
The effect of ALA on biochemical parameters of control and diabetic rats, 4 and 8 weeks after diabetes induction. Serum concentrations of glucose (a), triglycerides (b), urea (c), and creatinine (d). Values are mean ± SD. “^∗^” indicates statistically significant difference compared to the control group (^∗^*p* < 0.05, ^∗∗^*p* < 0.01, and ^∗∗∗^*p* < 0.001), “^#^” indicates statistically significant difference compared to the ALA group (^#^*p* < 0.05, ^##^*p* < 0.01, and ^###^*p* < 0.001), and “*δ*” indicates statistically significant difference compared to the STZ group of animals (*^δ^p* < 0.05, *^δδ^p* < 0.01, and *^δδδ^p* < 0.001).

Glucose, triglycerides, urea, and creatinine were determined in serum of control, diabetic, and ALA-treated nondiabetic and diabetic rats (Figure 1). Diabetic condition was followed 4 weeks and 8 weeks, in order to include the time course necessary for detrimental processes to be manifested. Both groups of diabetic rats were characterized by hyperglycemia, increasing trends of triglycerides and creatinine level but without statistical significance and by significantly increased level of urea. In diabetic rats, 4 weeks of ALA treatment resulted in decrease of serum glucose level to the control value, while after 8 weeks glucose level declined but still remained significantly elevated compared to controls. In both diabetic groups treated with ALA, triglycerides, and creatinine were not significantly changed. Treatment with ALA significantly decreased urea level of diabetic rats after 4 weeks, while after 8 weeks urea serum level of diabetic rats decreased compared to diabetic controls but without statistical significance. Administration of ALA to control rats did not significantly affect the examined biochemical parameters.

### 3.2. Morphological Evaluation of Renal Structure and CML Formation after ALA Treatment

**Figure 2:**
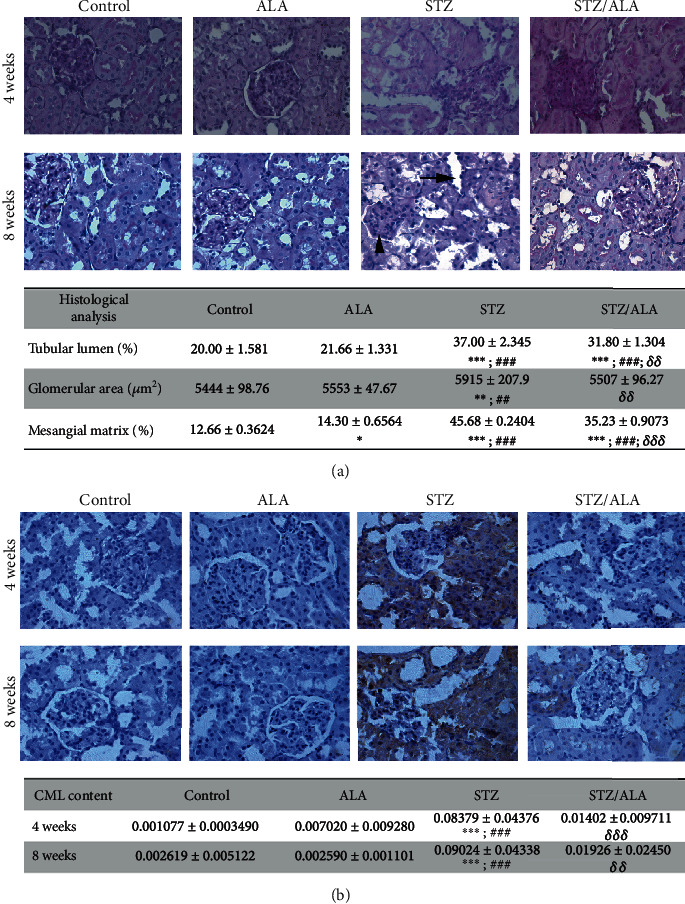
The effects of ALA on kidney structure and CML formation in control and diabetic rats, 4 and 8 weeks after diabetes induction. (a) Histological analysis of kidney structure and pathologic features associated with diabetes (tubular dilatation marked with arrow and mesangial matrix expansion with triangle) assessed by PAS staining. The results of histological analysis of kidney sections after 8 weeks of diabetes induction are presented as mean values ± SD for tubular lumen, glomerular, and mesangial matrix areas. (b) Immunohistochemical staining of carboxymethyllysine (CML) in the representative kidney section. The results of quantification by ImageJ analysis are presented as mean values ± SD. “^∗^” indicates statistically significant difference compared to the control group (^∗^*p* < 0.05, ^∗∗^*p* < 0.01, and ^∗∗∗^*p* < 0.001), “^#^” indicates statistically significant difference compared to the ALA group (^#^*p* < 0.05, ^##^*p* < 0.01, and ^###^*p* < 0.001), “*^δ^*” indicates statistically significant difference compared to the STZ group of animals (*^δ^p* < 0.05, *^δδ^p* < 0.01, and *^δδδ^p* < 0.001).

PAS staining highlighted the characteristic renal histopathologic features associated with diabetes (Figure 2(a)). Thickening of glomerular basement membrane appeared after 4 weeks of diabetes onset while mesangial matrix expansion and tubules with widened lumen were observed after 8 weeks of diabetes onset. Histological analysis revealed that the tubular lumen, glomerular, and mesangial matrix areas significantly increased in 8 weeks diabetic rats compared to control and ALA-treated control rats. However, after ALA treatment, tubular dilatation and mesangial matrix expansion significantly decreased but still remained above the control values, while the glomerular area was reverted completely to control levels. In control rats treated with ALA the mesangial matrix fraction was found to be increased suggesting that long-term use of ALA could affect some of the structural features in the kidneys (Figure 2(a)).

AGEs are pathogenic factors that contribute to renal damage in diabetes. In order to inspect whether ALA affects the accumulation of AGE products in the kidneys of diabetic rats, the CML renal content was assessed by immumohistochemical staining in control, diabetic rats (4 and 8 weeks) and ALA treated control and diabetic rats (4 and 8 weeks) (Figure 2(b)). Staining for CML was positive in both groups of diabetic rats revealing that CML accumulates mainly in the tubulointerstitium and to a lesser extent in the glomeruli. According to quantification of CML content, these changes were statistically significant compared to corresponding controls (Figure 2(b)). After ALA treatment of diabetic rats (4 and 8 weeks), both tubulointerstitial and glomerular CML immunoreactivity markedly decreased while in control rats treated with ALA, CML positivity remained undetectable as in controls.

### 3.3. The Effect of ALA on Collagen Deposition and Col4a1 Gene Expression

**Figure 3:**
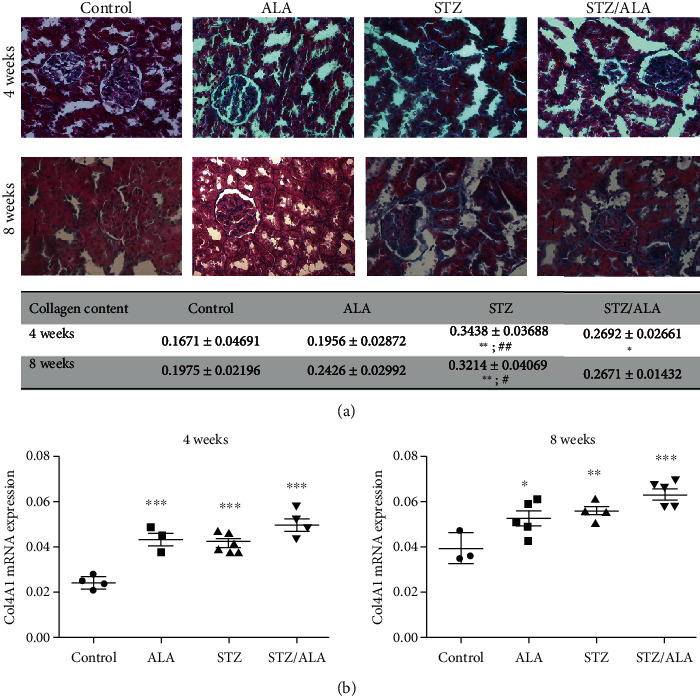
The effect of ALA on collagen deposition and *Col4a1* gene expression in kidneys of the control and diabetic rats, 4 and 8 weeks after diabetes induction. (a) Masson trichrome staining showing collagen deposition in kidney sections (blue: collagen, pink/violet: cytoplasm, and brown: nuclei). The results of quantification by ImageJ analysis are presented as mean values ± SD. (b) mRNA levels of *Col4a1* expression in the kidneys, as determined by RT-qPCR. mRNA levels are relative to *Actb*. “^∗^” indicates statistically significant difference compared to the control group (^∗^*p* < 0.05, ^∗∗^*p* < 0.01, and ^∗∗∗^*p* < 0.001), “^#^” indicates statistically significant difference compared to the ALA group (^#^*p* < 0.05, ^##^*p* < 0.01, and ^###^*p* < 0.001).

Masson trichrome staining revealed collagen deposition in tubulointerstitial areas and within glomeruli, in particular in basement membranes of both compartments in diabetic (4 and 8 weeks) groups of rats (Figure 3(a)). ALA treatment (4 and 8 weeks) of nondiabetic rats was also associated with increased collagen deposition characterized by glomerular and tubulointerstitial areas occupied by the collagen stained matrix but without statistical significance. In ALA-treated diabetic rats (4 and 8 weeks), the collagen deposition persistently remained since a significant decrease was not detected compared to diabetic rats. To verify increase in collagen synthesis, the level of *Col4a1* gene expression was assessed by RTqPCR (Figure 3(b)). A significant increase in *Col4a1* gene expression in the kidneys of diabetic rats in both time points, as well as in kidneys of ALA-treated diabetic and nondiabetic rats from both experimental groups (4 and 8 weeks) was detected, compared with respective controls.

### 3.4. Profibrotic Changes Associated with Diabetes and ALA Treatment

**Figure 4:**
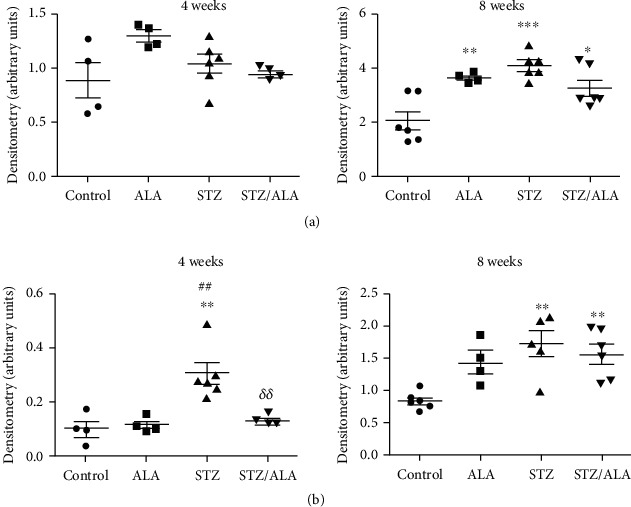
Quantification of TGF-*β*1 (a) and *α*SMA (b) protein levels of kidney homogenates from the ALA-treated or nontreated, control, and diabetic rats, 4 and 8 weeks after diabetes induction, detected by Western blot analysis. The densities of the bands from three immunoblots (representative blots shown in Supplementary material, Figure S1) are presented as the mean ± SD after normalization to GAPDH. “^∗^” indicates statistically significant difference compared to the control group (^∗^*p* < 0.05, ^∗∗^*p* < 0.01, and ^∗∗∗^*p* < 0.001), “^#^” indicates statistically significant difference compared to the ALA group (^#^*p* < 0.05, ^##^*p* < 0.01, and ^###^*p* < 0.001), “*^δ^*” indicates statistically significant difference compared to the STZ group of animals (*^δ^p* < 0.05, *^δδ^p* < 0.01, and *^δδδ^p* < 0.001).

Western blot analysis revealed that TGF-*β*1 protein level did not vary significantly after 4 weeks regardless of treatment (Figure 4(a)). However, statistically significant induction of TGF-*β*1 protein was detected after 8 weeks of diabetes, which was slightly reduced by ALA treatment but remained significantly increased compared with control. Moreover, similar induction of TGF-*β*1 was observed in nondiabetic animals treated with ALA (Figure 4(a)). To examine whether TGF-*β*1 induction was accompanied with the occurrence of fibrotic markers, the presence of *α*SMA was analyzed using Western blot (Figure 4(b)). Diabetic state triggered *α*SMA expression that was detected from the fourth week after diabetes onset and onwards. ALA treatment of diabetic rats reduced *α*SMA to the control level after 4 weeks but was ineffective after 8 weeks. ALA treatment did not affect *α*SMA in nondiabetic rats after 4 weeks, while after 8 weeks, it led to augmented *α*SMA expression in respect to control, but this increase was not statistically significant (Figure 4(b)).

Treatment of nondiabetic rats with ALA resulted in similar imprint regarding profibrotic features compared to diabetic rats, indicating need for unveiling the mechanisms that underlie the observed fibrotic-like changes in the kidneys of nondiabetic rats treated with ALA. In order to investigate the possibility that epigenetic modifications represent additional mechanism involved in the observed ALA-induced profibrotic landscape in nondiabetic rats, in particular increase of *Col4a1* expression, kidneys from control and ALA-treated nondiabetic rats were further subjected to DNA methylation status analysis.

### 3.5. The Impact of ALA on DNA Methylation Status

**Figure 5:**
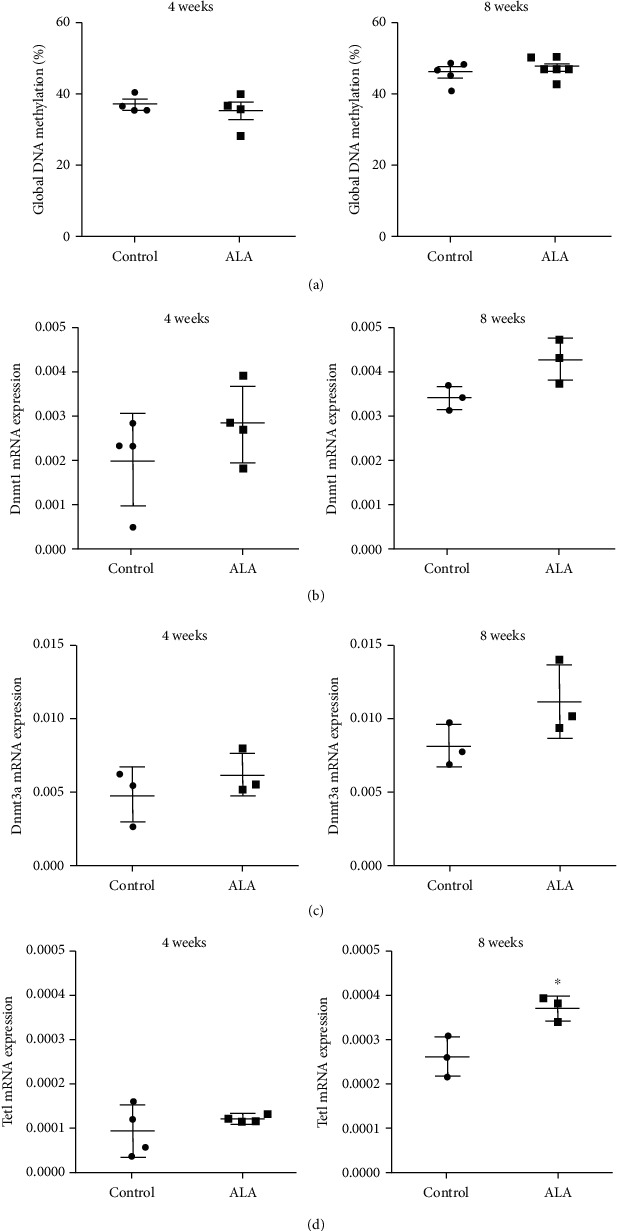
The impact of ALA on DNA methylation status estimated in the kidneys of control rats after 4 and 8 weeks of treatment. (a) Global level of DNA methylation measured by ELISA-based assay. Levels of mRNA of Dnmt1 (b), Dnmt3a (c), and Tet1 (d) expressions in the kidneys as determined by RT-qPCR. mRNA levels are relative to Actb. ^∗^*p* < 0.05 compared to the control group of animals.

The global level of DNA methylation and gene expression of the major players involved in DNA methylation (*Dnmt1*, *Dnmt3a*, and *Dnmt3b)* and demethylation (*Tet1)* were assessed in kidneys of nondiabetic control and ALA-treated rats for 4 and 8 weeks (Figure 5). Only *Tet1* mRNA level exhibited a statistically significant increase after 8 weeks of ALA treatment, whereas the expression of *Dnmt*s and global level of DNA methylation did not show a significant difference neither after 4 nor 8 weeks of ALA treatment compared to controls.

**Figure 6:**
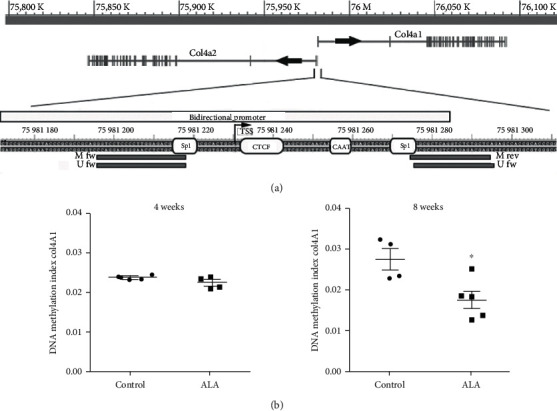
The influence of ALA treatment on DNA methylation status of the *Col4a1*/*Col4a2* promoter region. (a) Schematic representation of genomic loci that encompasses *Col4a1* and *Col4a2* genes and enlarged region of their bidirectional promoter (130 bp long) with marked position of *Col4a1* transcription start site (+1), positions of primers used for methylation-specific PCR, and transcription factors binding sites. (b) DNA methylation levels of the selected region of *Col4a1*/*Col4a2* promoter obtained by MSP and estimated as DNA methylation index. ^∗^*p* < 0.05 compared to the control group of animals.

The next experiment was directed to examination of local level of DNA methylation of *Col4a1* gene. The major form of collagen IV contains two *α*1(IV) chains and one *α*2(IV) chain, encoded by *Col4a1* and *Col4a2* genes which are arranged in head to head orientation forming a unique transcriptional unit, and transcribed in different directions from the mutual promoter [17]. *Col4a1* is transcribed from the 130 bp long intergenic region containing two Sp1 sites, CAAT and CTC boxes [17](Figure 6(a)). DNA methylation status of *Col4a1/Col4a2* promoter was assessed using MSP analysis and position of primers is shown on Figure 6(a). Both primer pairs, specific for methylated and for unmethylated DNA, covered 2 CpGs per primer. Results of MSP revealed that ALA did not affect methylation status of *Col4a1/Col4a2* promoter after 4-week-long treatment (Figure 6(b)). However, statistically significant decrease in DNA methylation index was observed after 8 weeks of ALA treatment (Figure 6(b)), suggesting potential of ALA to modulate *Col4a1* expression throughout alteration of DNA methylation pattern of its promoter.

## 4. Discussion

Due to epidemic proportion of diabetes, DKD becomes the most frequent cause of end-stage renal disease worldwide and the most indicative factor for premature mortality in patients with diabetes [28, 29]. The incidence of DKD in diabetic patients has not been decreased in past decades in spite of intense treatment directed towards blood sugar control [28, 30]. Among potential treatment strategies that have shown benefit against DKD is oxidative stress targeting and in that term it was established that ALA can improve renal function in diabetes [29, 31, 32]. The results obtained in present study confirmed hypoglycemic effects of ALA, but indicated ineffectiveness of ALA against disturbed urea level in long-term diabetes.

Increase in glycemia alone or combined with oxidative stress is one of the mechanisms involved in the formation of AGEs whose accumulation in glomerular and tubulointerstitial compartments was correlated with the degree of the renal damage in experimental model system of diabetic nephropathy [33, 34]. CML is one of the major AGEs *in vivo* [35] whose level increases in circulation and organs including kidneys of diabetic patients [36], playing an important role in the pathogenesis of diabetic nephropathy [37]. Our results showed that CML deposition accumulates mainly in the tubulointerstitial and to a lesser extent in the glomerular compartment of diabetic rat kidney which markedly decreased after ALA treatment. According to published data, tubules enriched with CML could be the most seriously injured renal compartment [38–40]. In discrepancy with our result, limited effects of ALA on the CML content in diabetic rats, detected both by immunostaining and chemical analysis, have been described [40] and this inconsistency may arise from experimental design of the study.

Histological analysis of a kidney structure by PAS staining confirmed protective effects of ALA in diabetes. The beneficial antioxidant effects of ALA against diabetes-induced oxidative injury in kidneys were well-established [22, 31, 32]. Treatment with ALA was found to prevent renal insufficiency and to alleviate renal structural pathological changes including glomerular mesangial matrix expansion and glomerulosclerosis due to its combined antioxidant and hypoglycemic potential [21, 22]. Considering that high glucose, oxidative stress and formation of AGEs are acknowledged as important factors for the induction of profibrotic changes in the kidney [41], well-established antioxidant and hypoglycemic effects, and capability of ALA to reduce CML content strongly advocates for potential of ALA to alleviate fibrotic process in diabetic kidneys. However, our results revealed that in Masson's-stained sections of kidney tissue, the degree of collagen fiber deposition did not significantly changed after ALA treatment of diabetic rats. Moreover, ALA treatment showed the tendency to increase collagen synthesis/content in non-diabetic rats. In accordance with our findings, the detrimental effects of ALA on control/healthy kidney have been reported [42]. Studying the renoprotective mechanisms of ALA in diabetic nephropathy, the authors unexpectedly discovered that treatment with ALA was associated with development of glomerulosclerosis, tubulointerstitial fibrosis and decline in renal function in the nondiabetic kidneys. Independent studies revealed that higher doses of ALA provoke oxidative protein damage in aged rats [43] and even renal function decline in diabetes [40]. In line with this, the prooxidant effect of ALA was evidenced not only in animal studies but also in patients suffering from end-stage renal disease receiving intravenous iron [44].

In the context of the observed effects of ALA on collagen deposition, our results additionally showed that ALA did not reduced the level of two fibrotic markers, TGF-*β*1 and *α*-SMA, in diabetic rats after 8 weeks and even induced TGF-*β*1 expression in nondiabetic rats after 8 weeks of treatment. In line with that, the gene expression of collagen type IV was significantly higher in diabetic as well as in the ALA treated diabetic and nondiabetic rats compared to controls. Previously, it has been shown that oxidative stress induces TGF-*β*1 and its downstream target genes such as collagen types I, III, and IV, as well as fibronectin [9, 45] and that antioxidants repress TGF-*β*1 expression [46]. In contrast to general idea of protective and antifibrotic role of antioxidants, our results suggest that ALA could have multiple effects on factors involved in fibrotic process in diabetic kidneys. It seems that ALA specifically induces expression of some fibrosis-related genes such as *TGFB1* and especially *Col4a1* whose increased expression was detected already after 4 weeks of ALA treatment in diabetic and nondiabetic kidneys. Enhanced expression and deposition of collagen type I have been observed after ALA treatment in human dermal fibroblasts [24]. The study revealed that upregulation of collagen type I results from TGF-*β* receptor I downstream signaling, thus suggesting novel pharmacological effect of ALA involved in skin aging. Considering results presented herein, upregulation of collagens might be a common cell response to ALA rather than specific mechanism characteristic for dermal fibroblasts.

Collagen type IV synthesis is regulated on well-established transcriptional, posttranscriptional, and posttranslational level, but more data regarding the role of DNA methylation in *Col4a1* expression are emerging. Study performed 40 years ago revealed that treatment of F9 teratocarcinoma cells with DNA demethylating agent 5-Azacytidine results in increase *Col4a1* expression, suggesting for the first time that DNA methylation may play important role in its expression [47]. Human methylome and transcriptome studies revealed correlation of *Col4a1* promoter methylation with its expression in healthy and liver cells activated to produce ECM proteins [20, 48]. The same was found in mesangial cells where following 5-Azacytidine treatment, *Col4a1* mRNA levels increased significantly [49]. Moreover, in patients with chronic kidney disease, DNA methylation level of *Col4a1* and *Col4a2* genes was found to be significantly lower compared to healthy subjects and in correlation with increased mRNA and protein expressions [50]. Our results confirmed strong evidence of regulation of *Col4a1* expression through DNA methylation as we found that *Col4a1* expression correlates with methylation status of its promoter in kidney. It should be noted that the observed changes in methylation status of *Col4a1* promoter most probably affect also the expression of *Col4a2*, as two genes are regulated via mutual bidirectional promoter region. MSP primers used in our experiments were designed to overlap partially with two Sp1 binding sites and decrease in DNA methylation that we observed after ALA treatment could reflect on improved binding of Sp1 and upregulation of *Col4a1* expression.

Our findings that *Col4a1* expression and its methylation status are prone to ALA action in nondiabetic animals after 8 weeks of ALA treatment additionally suggests potential of ALA to act as epigenetic regulator as well. According to our results, ALA affected the *Tet1* enzyme after 8 weeks of treatment, towards its increased expression which indicates possibility that gene specific demethylation could occur with no detectable net-effect on genome wide DNA methylation. Although there is a correlation between increased expression of *Col4a1* and decreased methylation of its promoter after 8 weeks of ALA treatment, still it is difficult to consider unequivocally that DNA demethylation is the only causal link between ALA treatment and increased collagen gene expression given that at 4 weeks, increased expression of *Col4a1* was not associated with alteration in its methylation status. The ability of ALA to regulate certain genes epigenetically has been proposed for the *IL-1β* and *IL-6* genes whose down-regulatated mRNA and protein levels were associated with hypermethylation of their promoter regions [51, 52]. Though hypermethylation was proposed as one of the mechanisms of ALA action, detailed pathways of epigenetic regulation driven by the ALA has not been elucidated yet and wait to be confirmed for the other genes as well. Hence, the link between increased collagen synthesis/deposition that may result in pathological phenotype and Tet-mediated DNA demethylation of *Col4a1* promoter provoked by the ALA treatment requires additional proving and should provide the platform for the future studies.

## 5. Conclusions

The protective role of ALA against diabetes-related malfunctioning of different organs including the kidneys has been mostly attributed to its antioxidant and hypoglycemic effects. However, the results presented in this study suggest that renal protection in diabetes by ALA could be limited due to increase of collagen synthesis/deposition in the kidneys. By analyzing the effects of ALA on profibrotic processes in diabetic rat kidneys, we found that ALA did not reduce either collagen synthesis or profibrotic markers. Moreover, treatment with ALA contributed to profibrotic events and collagen synthesis even in nondiabetic rat kidneys that is partially related to changes in DNA methylation status of *Col4a1* gene. Consequently, in patients with renal fibrosis, the caution regarding the use of ALA is needed in order to avoid the aggravation of the existing renal complication. Moreover, the caution is needed when ALA is to be used for boosting antioxidant potential in healthy person or in the management of other diabetic complications as beneficial effects for one complication could be detrimental for the other.

## Data Availability

All relevant data used to support the findings of this study are included within the article.

## Supplementary Materials

Supplementary Materials Supplementary Table S1: Sequences of primers used for real-time quantitative PCR analysis of *Col4a1* (Collagen type IV alpha 1 chain)*, Dnmt1* (DNA methyltransferase 1)*, Dnmt3a* (DNA methyltransferase 3 alpha)*, Dnmt3b* (DNA methyltransferase 3 beta)*, Tet1* (Tet methylcytosine dioxygenase 1) and *Actb* (Actin, beta) gene expression. Supplementary Table S2: Sequences of primers for MSP analysis of DNA methylation of promoter region of *Col4a1* gene. Supplementary Figure S1: Representative images of immunoblotting. Protein levels of TGF-*β*1 (A) and *α*SMA (B), two fibrosis-related proteins, in kidneys of ALA treated or non-treated, control and diabetic rats, 4 and 8 weeks after diabetes induction, determined by Western blot analysis.
