# Supplementary material for: α-Lipoic Acid Increases Collagen Synthesis and Deposition in Nondiabetic and Diabetic Rat Kidneys
Source: Oxid Med Cell Longev. 2021 Mar 11;2021:6669352. doi: 10.1155/2021/6669352 (PMC7979310; doi:10.1155/2021/6669352)

**α-Lipoic Acid Increases Collagen Synthesis and Deposition in Non-Diabetic and Diabetic Rat Kidneys**

**SUPPLEMENTARY MATERIAL**

**Table S1.** Sequences of primers used for real-time quantitative PCR analysis of gene expression.

| **Name** | | **5'-3' sequence** |
| --- | --- | --- |
| *Col4a1* | fw | GGGAGATCAAGGCATACCCG |
|  | rev | ACCATCAAACCCAGGGACAC |
| *Dnmt1* | fw | GCTAAGGACGATGATGAGAGC |
|  | rev | CTTTTTGGGTGACGGCAACTC |
| *Dnmt3a* | fw | CAGCGTCACACAGAAGCATATCC |
|  | rev | GGTCCTCACTTTGCTGAACTTGG |
| *Dnmt3b* | fw | ACCTGGAAGAGTTTGAGCCG |
|  | rev | GAAGAATGGACGGTTGTCGC |
| *Tet1* | fw | CCTGTCAATGTGCTCGTCCT |
|  | rev | TAATCACCCACTTGGCGACC |
| *Actb* | fw | AGATTACTGCCCTGGCTCCT |
|  | rev | ACATCTGCTGGAAGGTGGAC |

**Table S2.** Sequences of primers used for MSP analysis of DNA methylation.

| **Name** | | | Sequence 5'-3' |
| --- | --- | --- | --- |
| *Col4a1* | M | fw | AATCCTCCCCTTAAAAACGCC |
|  |  | rev | GAGTAGAGATTTGAGCGCGT |
|  | U | fw | AATCCTCCCCTTAAAAACACC |
|  |  | rev | GTTTTGAGTAGAGATTTGAGTGTG |

**Figure S1.** Representative images of immunoblotting

Protein levels of TGF-β1 (A) and αSMA (B), in kidneys of ALA treated or non-treated, control and diabetic rats, 4 and 8 weeks after diabetes induction determined by Western blot analysis.


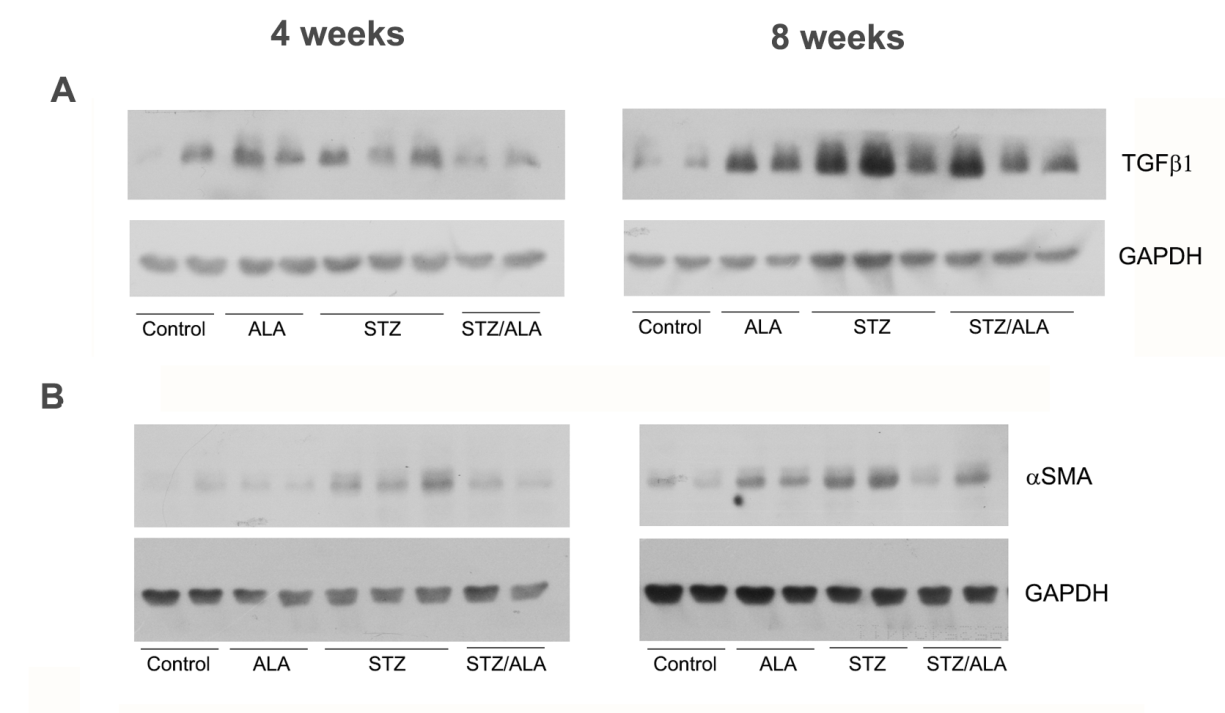

Supplement: Supplementary Materials — Supplementary Table S1: Sequences of primers used for real-time quantitative PCR analysis of Col4a1 (Collagen type IV alpha 1 chain), Dnmt1 (DNA methyltransferase 1), Dnmt3a (DNA methyltransferase 3 alpha), Dnmt3b (DNA methyltransferase 3 beta), Tet1 (Tet methylcytosine dioxygenase 1) and Actb (Actin, beta) gene expression. Supplementary Table S2: Sequences of primers for MSP analysis of DNA methylation of promoter region of Col4a1 gene. Supplementary Figure S1: Representative images of immunoblotting. Protein levels of TGF-β1 (A) and αSMA (B), two fibrosis-related proteins, in kidneys of ALA treated or non-treated, control and diabetic rats, 4 and 8 weeks after diabetes induction, determined by Western blot analysis. [file 6669352.f1.docx]
